# The positive piezoconductive effect in graphene

**DOI:** 10.1038/ncomms9119

**Published:** 2015-09-11

**Authors:** Kang Xu, Ke Wang, Wei Zhao, Wenzhong Bao, Erfu Liu, Yafei Ren, Miao Wang, Yajun Fu, Junwen Zeng, Zhaoguo Li, Wei Zhou, Fengqi Song, Xinran Wang, Yi Shi, Xiangang Wan, Michael S. Fuhrer, Baigeng Wang, Zhenhua Qiao, Feng Miao, Dingyu Xing

**Affiliations:** 1 National Laboratory of Solid State Microstructures, School of Physics, Collaborative Innovation Center of Advanced Microstructures, Nanjing University, Nanjing 210093, China; 2 ICQD, Hefei National Laboratory for Physical Sciences at Microscale, and Synergetic Innovation Center of Quantum Information and Quantum Physics, University of Science and Technology of China, Hefei, Anhui 230026, China; 3 Key Laboratory of Strongly-Coupled Quantum Matter Physics, Chinese Academy of Sciences, and Department of Physics, University of Science and Technology of China, Hefei, Anhui 230026, China; 4 Department of Physics, University of Maryland, College Park, Maryland 20742, USA; 5 State Key Laboratory of ASIC and System, Department of Microelectronics, Fudan University, Shanghai 200433, China; 6 School of Electronic Science and Engineering, Nanjing University, Nanjing 210093, China; 7 School of Physics, Monash University, Monash, Victoria 3800, Australia

## Abstract

As the thinnest conductive and elastic material, graphene is expected to play a crucial role in post-Moore era. Besides applications on electronic devices, graphene has shown great potential for nano-electromechanical systems. While interlayer interactions play a key role in modifying the electronic structures of layered materials, no attention has been given to their impact on electromechanical properties. Here we report the positive piezoconductive effect observed in suspended bi- and multi-layer graphene. The effect is highly layer number dependent and shows the most pronounced response for tri-layer graphene. The effect, and its dependence on the layer number, can be understood as resulting from the strain-induced competition between interlayer coupling and intralayer transport, as confirmed by the numerical calculations based on the non-equilibrium Green's function method. Our results enrich the understanding of graphene and point to layer number as a powerful tool for tuning the electromechanical properties of graphene for future applications.

## Abstract (web summary)

The thinness and elastic properties of graphene make it an ideal candidate for nanoelectromechanical systems. Here, the authors demonstrate the positive piezoconductive effect in suspended bi- and multi-layer graphene, showing the most pronounced response for three layer samples.

## Graphical abstract

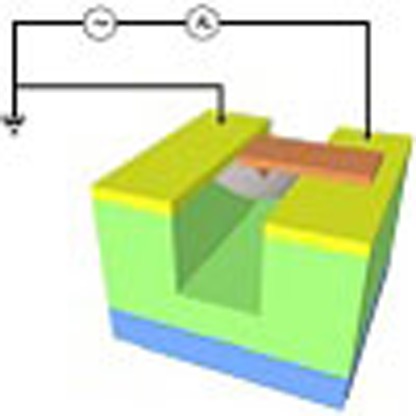

Graphene^1^,^2^,^3^,^4^ is an ideal material candidate for nano-electromechanical systems^5^ due to many advantageous features, including unparalleled breaking strength^6^, ultrahigh carrier mobility^7^ and excellent controllability of electronic structures via mechanical strain^8^,^9^. Many intriguing phenomena have been experimentally observed on strained graphene^10^,^11^,^12^, including the observation of pseudo-magnetic fields exceeding 300 T (ref. 13). More fascinating phenomena have been theoretically predicted for strained graphene, but yet to be realized experimentally, such as the zero-field quantum Hall effect^14^, strain-induced superconductivity of graphene^15^ and potential applications in valleytronics^16^,^17^. Thus, strain-controllable transport measurements are critical for in-depth understanding and further applications of graphene.

*In situ* piezoconductive measurements on graphene provide an effective approach to study the correlations between electrical properties and mechanical strains. So far, studies have been focused on mono-layer graphene, and the negative piezoconductive effect has been widely reported, independent of the differences in graphene film synthesis, transfer methods and sample substrates^18^,^19^,^20^,^21^,^22^,^23^. Systematic studies of the electromechanical properties of graphene with different number of layers and alterable interlayer interactions have been lacking.

Here we investigate the piezoconductive effect of suspended graphene membranes with various layer numbers by applying *in situ* stress with a scanning probe. We observe positive piezoconductance in bi- and multi-layer graphene, with tri-layer graphene showing the most pronounced response. This intriguing phenomenon can be explained by the model of strain-induced competition between electronic interlayer coupling and intralayer transport, and further confirmed by numerical calculations based on non-equilibrium Green's function method.

## Results

### Device fabrication and piezoconductive measurements

**Figure 1:**
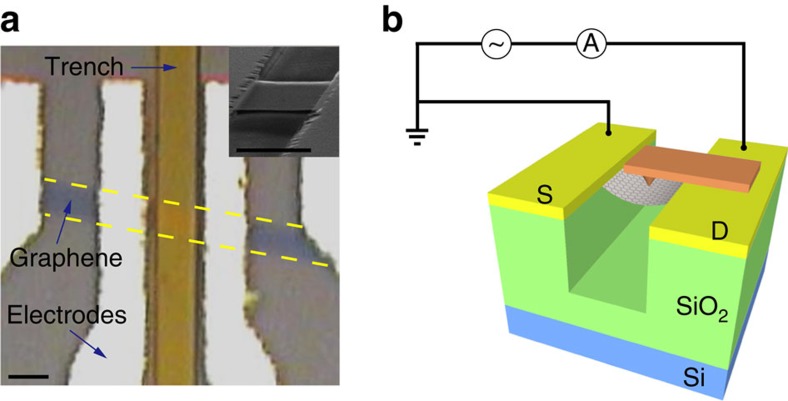
Suspended graphene device and PCM setup. (**a**) Optical microscope image of a four-terminal suspended bilayer graphene device, which is fabricated by home-made shadow mask method. Inset: SEM image of a suspended device. Scale bar, 3 μm. (**b**) Schematic setup of pressure-modulated conductance microscopy (PCM) that performs piezoconductive measurements on suspended graphene devices.

Suspended graphene membranes are mechanically exfoliated and deposited on Si/SiO_2_ wafers with pre-etched trenches. The number of the graphene layers is identified via colour interference, and confirmed by Raman spectroscopy. Metal electrodes (5 nm Ti/50 nm Au) are deposited through home-made shadow masks, which effectively avoid wet process-induced device performance degradation^24^. A typical device image is shown in Fig. 1a. To perform *in situ* piezoconductive measurements, we introduce pressure-modulated conductance microscopy^25^,^26^, which utilizes a non-conducting atomic force microscopy (AFM) tip to apply adjustable local pressure on the top of the suspended graphene, yielding a topography image of the strained graphene. The conductance/resistance of the device is monitored simultaneously, and comparison of the conductance/resistance image and topography offers piezoconductive information of the suspended graphene membranes. The detailed experimental setup is schematically shown in Fig. 1b.

**Figure 2:**
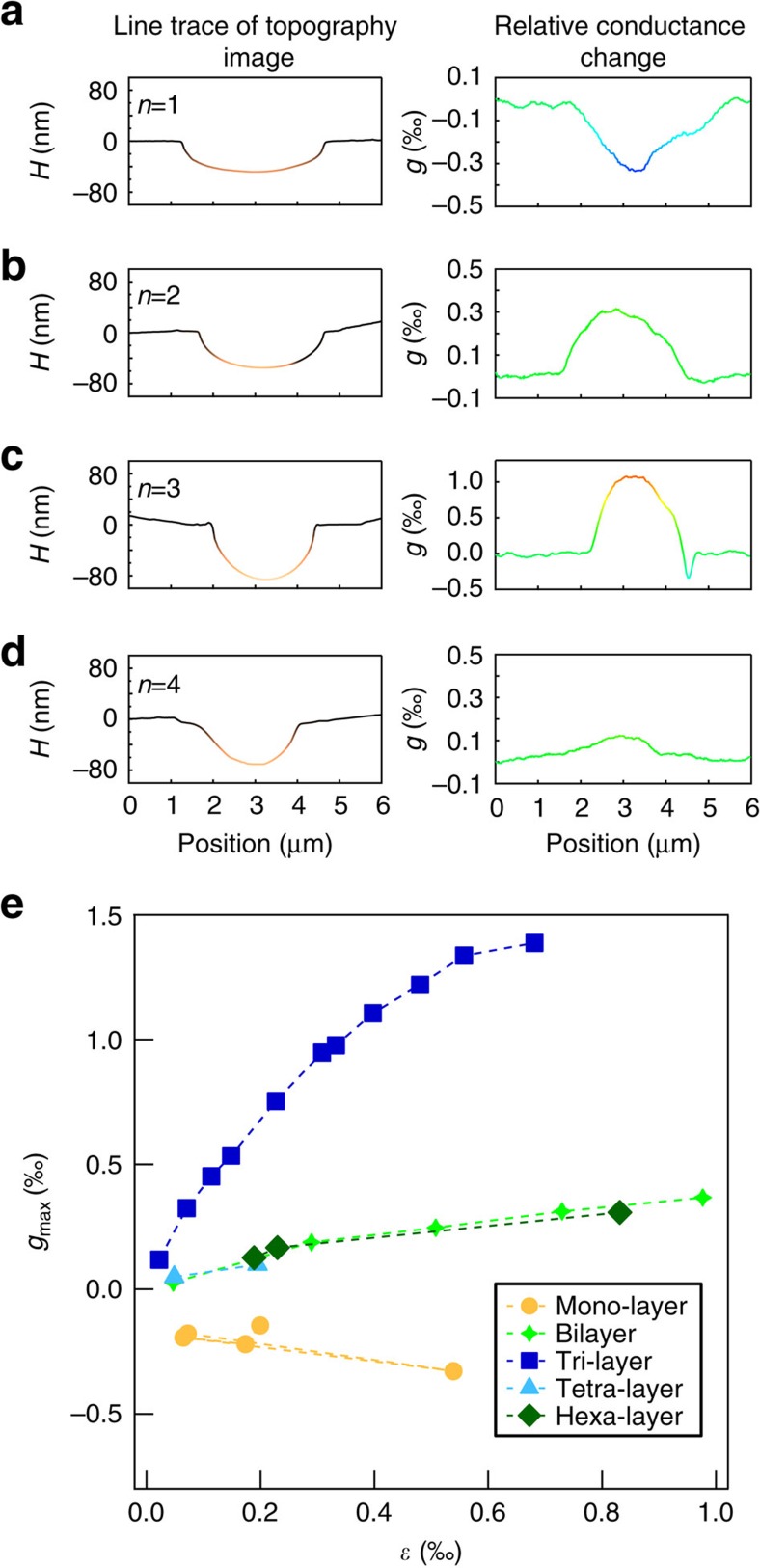
Layer number-dependent positive piezoconductive effect of graphene. (**a**–**d**) Left panels are the line traces of the topography (tip position) image of graphene devices from mono-layer (**a**) to tetra-layer (**d**) with applied strain *ɛ*=0.54‰, 0.51‰, 0.33‰ and 0.20‰, respectively. Right panels are the line traces of the corresponding relative conductance change *g* (to the undisturbed conductance with no local pressure applied). Mono-layer device shows negative piezoconductive effect (conductance drops upon local pressure applied). Bi-, tri- and tetra-layer devices show positive piezoconductive effect (conductance jumps upon local pressure applied) with the most pronounced effect in tri-layer device. (**e**) The plot of the maximum relative conductance change (when AFM tip approaches the centre of the suspended membranes) *g*_max_ as a function of strain *ɛ* for various suspended graphene devices (layer number, *n*=1, 2, 3, 4 and 6 represented by different colours).

The measurements are carried out on mono- and multi-layer suspended graphene devices. Typical piezoconductive results from mono-, bi-, tri- and tetra-layer devices are shown in Fig. 2a–d, respectively. For each figure, the left panel shows a line trace of the topography image and the right panel shows the corresponding relative conductance change represented by *g*. Here *g*(*x*) is defined by
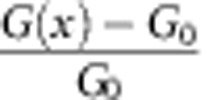
, where *G*(*x*) is the device conductance at AFM tip position *x* (where local pressure is applied) and *G*_0_ is the undisturbed conductance value. For mono-layer suspended graphene, the device conductance drops upon local pressure applied (Fig. 2a), indicating negative piezoconductive (that is, positive piezoresistive) effect. This is consistent with all previous studies^19^,^20^. However, for multi-layer suspended graphene, device conductance jumps upon local pressure applied (Fig. 2b–d), indicating positive piezoconductive effect, which has never been reported on graphene devices. We note that the measured tri-layer graphene device is stacked with the common Bernal (ABA) structure, as confirmed by Raman spectroscopy.

To further study the observed layer number-dependent piezoconductive effect, we perform the same measurement on various suspended graphene devices (layer number, *n*=1, 2, 3, 4 and 6) with different strains (up to 1‰). The detailed results are shown in Fig. 2e. Here we plot the maximum relative conductance change *g*_max_ (which usually appears when AFM tip approaches the centre of the suspended membranes). According to the geometry of our devices, the strain *ɛ* can be calculated by the equation


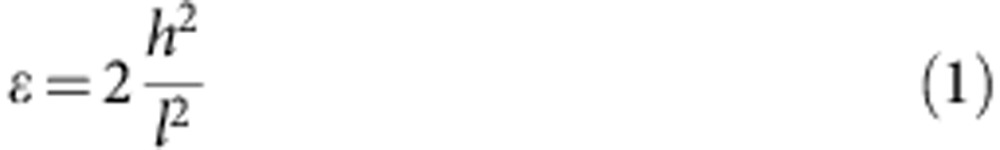


where *h* is the maximum strain-induced deflection and *l* is the length of the suspended graphene (same as the width of the trench, around 3 μm). Here *h* has been corrected by subtracting the height of the tip at which the force begins to rise (which can be extracted from the deflection-force curve, see Supplementary Fig. 1 and Supplementary Note 1 for details). As shown in the *g*_max_ versus *ɛ* plot in Fig. 2e, several data points from a mono-layer graphene device fall in the negative regime. The gauge factor (usually defined by relative resistance change divided by strain) is estimated to be about 0.6, similar to the values previously reported^19^,^20^. In sharp contrast, all multi-layer graphene devices show positive conductance response. More interestingly, for similar strain value, the *n*=3 (tri-layer) device shows much larger *g*_max_ than the other multi-layer devices (*n*=2, 4 and 6).

### Theoretical interpretation of the underlying physical origin

The positive piezoconductive effect is difficult to explain in terms of strain-induced decrease of Fermi velocity, the model that has been applied to strained mono-layer graphene^19^,^20^. In the system of back-gated suspended mono-layer graphene, a model of inhomogeneous carrier density redistribution predicted a positive piezoconductive effect^27^ in contrast to our observation of negative piezoconductance for mono-layer graphene, and cannot explain our observation as well. The fact that positive piezoconductance is only observed in bi- and multi-layer graphene suggests that interlayer coupling is key; indeed, strain should modify the interaction between graphene layers, modifying the band structure and hence transport properties.

To understand the observed positive piezoconductive effect, we numerically calculate the transport properties of strained multi-layer graphene devices, which can be calculated by applying non-equilibrium Green's function technique and using two-terminal Landauer–Büttiker formula, with details described in the Methods section. To numerically study the piezoconductive effect in the multi-layer graphene systems in the presence of an external pressure, we consider a *π*-orbital tight-binding model Hamiltonian, which is written as^4^,^28^,^29^:


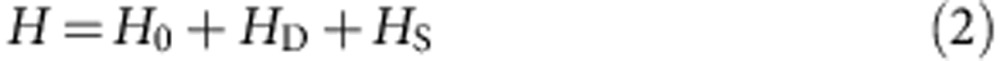



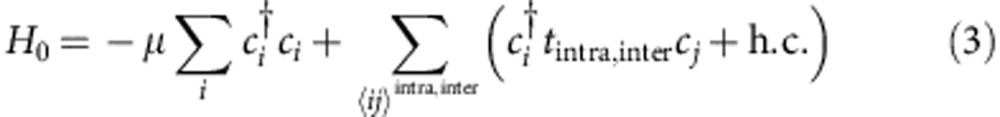



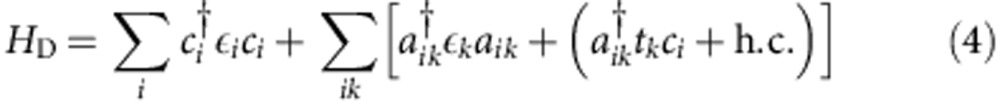



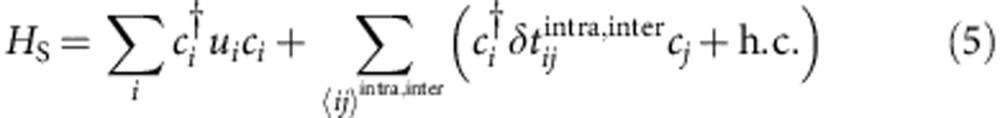


where 
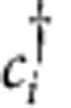
 and *c*_*i*_ are the creation and annihilation operators on the site *i*. The first term *H*_0_ describes the pristine multi-layer graphene sheets, with *μ* being the chemical potential and *t*_intra,inter_ denoting the intra- and interlayer nearest-neighbour hopping strength, respectively. In our consideration, we take *t*_intra_ =2.60 eV and *t*_inter_ =0.34 eV, respectively. The second term *H*_D_ describes the influences of external disorders and the dephasing effect that is used to recover the macroscopic behaviour from a finite-sized quantum system and can be modelled by attaching individual virtual leads at each site *i*. Here *ɛ*_*i*_ is the on-site Anderson disorder strength that is uniformly distributed in the interval of (−*W*/2, *W*/2) with *W* characterizing the strength of disorder and 
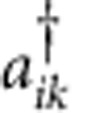
 and *a*_*ik*_ are the creation and annihilation operators for the virtual lead attached at the *i*-th site. In our consideration, we set the disorder strength to be 0.1 eV, because the prepared multi-layer graphene sheets are rather clean and it can better smear off the room temperature-induced thermal fluctuation. The last term *H*_S_ represents the applied strain-induced effects including the induced site potentials and the variation of the intra- and interlayer nearest-neighbour hopping energies in the lattice-deformed region. The AFM tip-contacted lattices should experience noticeable lattice deformations, while the lattice deformation farther away from the AFM tip gradually decreases. Thus it is reasonable to assume that the variation of the intra- and interlayer hopping energy due to the lattice deformation takes the form of 
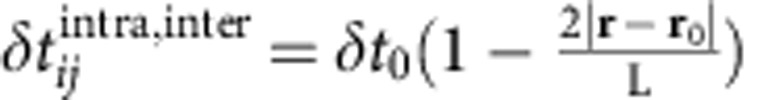
, where **r**_0_ is the centre of deformed region and L is the system length of the disordered scattering region, and the varying site potential takes the similar form of 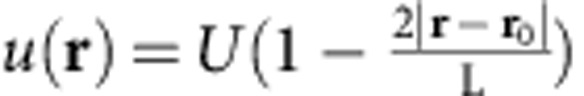. In our calculations, the conductance is averaged over 1,000 ensembles at each point.

**Figure 3:**
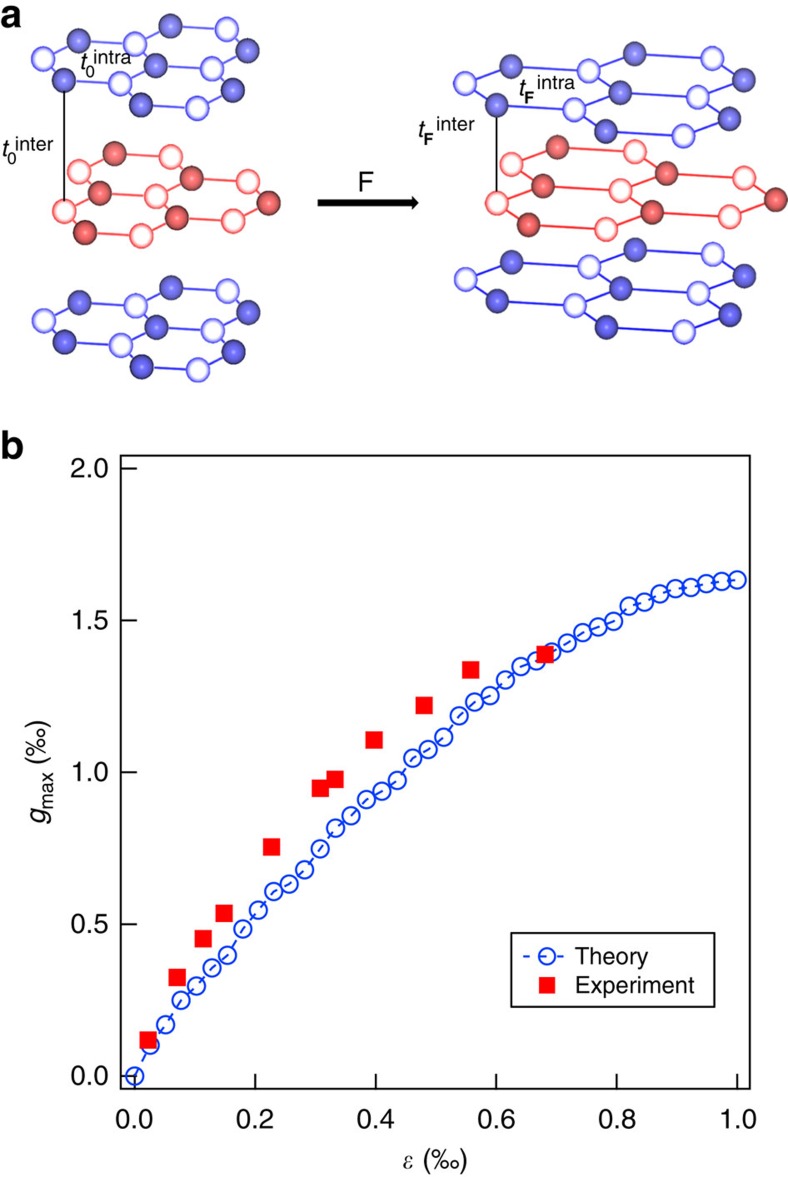
Theoretical model and numerical calculations of tri-layer graphene. (**a**) Schematic of the lattice structure change of Bernal (ABA) stacking tri-layer graphene due to a vertical load **F** applied. (**b**) The maximum relative conductance change *g*_max_ as a function of strain *ɛ* for tri-layer graphene. The red squares are the experimental data while the blue cycles are the numerical simulation results.

We first focus on the tri-layer graphene that shows the most pronounced positive piezoconductive effect. As illustrated in Fig. 3a, the tri-layer structure is constructed by mono-layer graphene in a Bernal ABA-stacking manner. Its conductance includes contributions from electron transport in both horizontal and vertical directions, and *g*_max_ is determined by the strain-induced competition between the intralayer hopping and interlayer interactions. In the presence of an applied pressure from the AFM tip, the direct consequences on the multi-layer graphene include: (1) the extension of the lattice constant in the lattice-deformed area that correspondingly decreases the intralayer nearest-neighbour hopping; and (2) the local compression of the interlayer separation in the lattice-deformed area that effectively modulates the interlayer interactions, that is, increasing the interlayer hopping and inducing the local site energies. By applying the non-equilibrium Green's function technique and Landauer–Büttiker formula (see details in the Methods section) on a tri-layer graphene ribbon with length of 100 nm and width of 13 nm, we theoretically study the relation between the maximum relative conductance change *g*_max_ and the applied strain *ɛ* for tri-layer graphene. It is noteworthy to mention that all our obtained data are used to qualitatively explain and understand the underlying physical origin of the positive piezoconductive effect, and for the better presentation we have chosen certain parameters to compare with the experimental observations. Our numerical results show that *g*_max_ is always positive and increases with increasing strain, as plotted in the blue cycles in Fig. 3b. The experimental data are plotted in the same graph, showing the similar tendency. The nonlinear feature indicates for higher strain *g*_max_ increases slower, and suggests the relative contribution from interlayer modulation becomes less dominant. This model also explains why the piezoconductive property of bi- and multi-layer graphene is distinct from mono-layer graphene. For strained mono-layer graphene, negative peizoconductance is observed because no interlayer modulation but only intralayer contribution is present.

## Discussion

**Figure 4:**
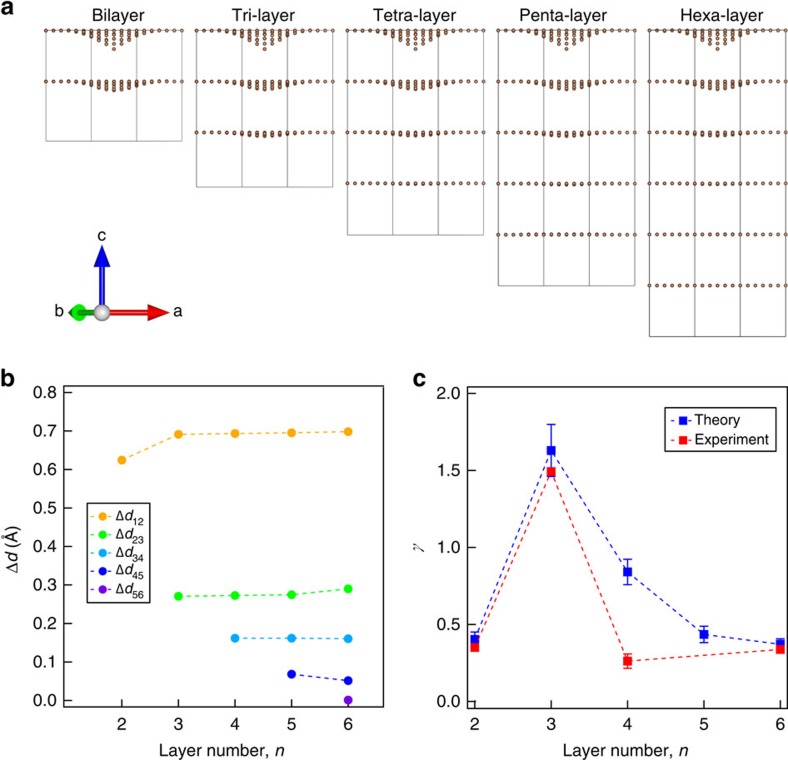
Simulation results of the dependence on layer number. (**a**) The structurally relaxed configuration for different multi-layer graphene (layer number, *n*=2–6 from left to right) in the presence of the same strain strength. (**b**) The strain-induced lattice variation Δ*d* between the nearest two layers as a function of the layer number *n*. (**c**) The dependence of the piezoconductive factor *γ* on layer number *n*, with experimental data represented by red squares and simulation results represented by blue squares. The error bars of experimental data originate from the fitting process, while the error bars of simulating results originate from different disorder configurations in the numerical calculations.

To further understand the intriguing phenomenon that tri-layer graphene shows the most pronounced positive piezoconductive effect, we have performed first-principle calculations to study the lattice configuration of different multi-layer graphene devices under strain. Our first-principle calculations are performed using the projected-augmented-wave method as implemented in the *Vienna ab initio simulation package*^30^ with details described in the Methods section. Figure 4a shows how the local lattice configuration is altered by the same local strain in different multi-layer graphene systems. For the bilayer graphene, one can observe that both the top and bottom layers exhibit the structural deformations with different amplitudes. We further plot the strain-induced lattice variation Δ*d* between the nearest two layers as a function of the layer number *n* in Fig. 4b. Along with the increase of the layer number, for example, from bilayer to up to hexa-layer, only the top three layers show noticeable structural deformations. Therefore, the positive piezoconductive effects for multi-layer graphene systems due to the interlayer modulations should be dominated by the top three layers.

We can now introduce a characteristic factor, the piezoconductive factor *γ*, to describe the piezoconductive properties of bi- and multi-layer graphene devices:


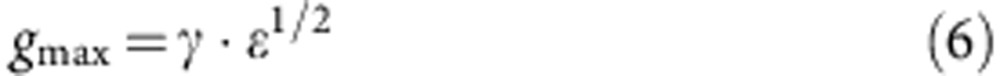


The piezoconductive factor *γ* has the same sign as piezoconductive effect. The square root dependence on strain is phenomenological but describes the experimental and theoretical data well and allows us to parameterize each *g*_max_(*ɛ*) curve by a single-value *γ*. By applying the same non-equilibrium Green's function technique as used in studying the tri-layer graphene, we calculate the *g*_max_–*ɛ* relation for graphene with various layer number *n* (*n*=2 to 6) (see detailed parameters in Supplementary Table 1), and extract the values of *γ* by using the above definition. The results are shown by the blue squares in Fig. 4c. The values of *γ* are positive, with the maximum value around 1.6 for tri-layer graphene. To compare the results with experimental observation, we plot the experimental data in the same graph (red squares) and find they are consistent with theoretical results. For bi- and tri-layer graphene, *γ* increases with *n*, suggesting the enhancement of the contribution from strain-induced interlayer modulation. But while *n* increases further, *γ* decreases, suggesting the interlayer modulation is dominated by the top three layers and additional parallel conduction paths from the extra layers suppress the positive piezoconductive effect. Here we note that, the roles of external effects (see Supplementary Figs 2–5 and Supplementary Note 2 for details), the stacking order (Supplementary Figs 6–8 and Supplementary Note 3) and back gate (Supplementary Fig. 9 and Supplementary Note 4), as well as finite size effect in the theoretical calculations (Supplementary Figs 10–11 and Supplementary Note 5), have been carefully explored in our work. Although the observation of negative piezoconductive effect in multi-layer graphene was reported previously^31^, it is likely related to the devices' unusually large contact resistance, rather than the intrinsic piezoconductive properties of multi-layer graphene as revealed in this manuscript.

In summary, we have systematically studied the piezoconductive effect of suspended graphene with different number of layers. In contrast to the negative piezoconductance observed in mono-layer graphene, positive piezoconductance is observed in bi- and multi-layer graphene, with tri-layer graphene showing the most pronounced response. It can be explained by the model of strain-induced competition between electronic interlayer coupling and intralayer transport. This model is further confirmed by numerical calculations based on non-equilibrium Green's function method. Our results enrich the understanding of electromechanical properties of graphene and underscore their potential applications on the field of nano-electromechanical systems and flexible electronics.

## Methods

### Suspended graphene device preparation and characterization

The suspended graphene membranes are obtained by using mechanical exfoliation method on the pre-defined trenches on 300-nm thick SiO_2_ wafers. The trenches are defined by standard photolithography method, followed by dry etching in an Inductively Coupled Plasma (ICP) system, where CH_4_ and CHF_3_ are used as etching gases. The typical width of the trenches is 3 μm and the depth is around 250 nm. The number of layers of graphene membranes is first identified by an optical microscopy and further confirmed by Raman spectroscopy. To avoid common wet process-induced device performance degradation and yield loss, the electronic devices are fabricated by using a home-made shadow mask method^24^. The electrodes are made of 5-nm Ti covered by 50-nm Au.

### Setup of pressure-modulated conductance microscopy

A Bruker Multimode 8 AFM is used to build up the pressure-modulated conductance microscopy setup. A non-conducting AFM tip is used to apply adjustable local pressure on the top of the suspended graphene, yielding topography/height image of the strained graphene. The conductance/resistance of the device is measured via lock-in technique and monitored simultaneously as an external input of the AFM. The comparison of the conductance/resistance image and topography image offers detailed piezoconductive information of the suspended graphene membranes. The scanning procedure is done in contact mode with a Bruker NP-S10 tip, of which the Young's modulus is 0.24 N m^−1^. The detailed experimental setup is schematically shown in Fig. 1b.

### Details of non-equilibrium Green's function simulation

Using multi-probe Landauer–Büttiker formula, the current in the lead *p* (either real or virtual lead) can be expressed as:


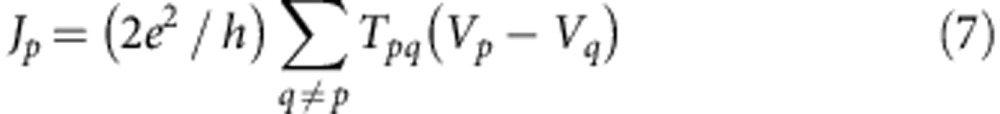


where *V*_*p/q*_ is the spin-independent bias in the lead *p*/*q*. The electronic transmission coefficient from lead *q* to lead *p* is calculated as *T*_*pq*_ = *Tr*[Γ_*p*_*G*^*r*^Γ_*q*_*G*^*a*^], in which the line-width function Γ_*p*_ is defined as 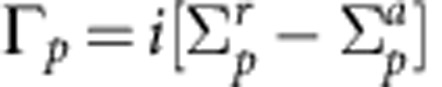, and the retarded and advanced Green's function are given by 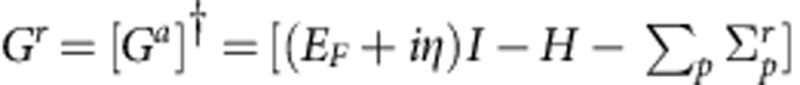, where *I* is the unit matrix with the same dimension as that of *H*. The retarded and advanced self-energy due to the coupling to all the real leads can be obtained numerically^32^. For the virtual leads, we assume 
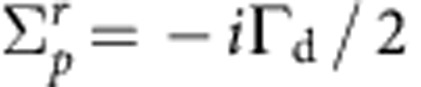
 and the dephasing strength Γ_d_ is fixed to 0.01 eV. In our simulations, a small external bias is applied between the left and right lead with *V*_L_=−*V*_R_=0.5 V. For dephasing effect, electrons lose the phase memory by entering and leaving the virtual leads. Thus, for each virtual lead *i*, the current has the constraint that *J*_*i*_=0, which ensures the current conservation. Combining the above equation of *J*_*p*_ together with all boundary conditions for the real and virtual leads, the voltage *V*_*p*_ and current *J*_*p*_ in each real lead can be obtained.

### Details of first-principle calculation

The generalized gradient approximation combined the Vander Waals correction with the DFT-D2 method of Grimme is used. The kinetic energy cutoff is set to be 500 eV. During the structure relaxation, the edge atoms and the probed atoms are not allowed to relax while the others are. All parameters are chosen to converge the forces to be <0.01 eV Å^−1^. The first Brillouin-zone integration is carried out by using the 3 × 3 × 1 Gamma-centred grids. A vacuum buffer space of 20 Å is set to prevent the interaction between adjacent slabs.

## Additional information

**How to cite this article:** Xu, K. *et al*. The positive piezoconductive effect in graphene. *Nat. Commun.* 6:8119 doi: 10.1038/ncomms9119 (2015).

## Supplementary Material

Supplementary Information Supplementary Figures 1-11, Supplementary Table 1, Supplementary Notes 1-5 and Supplementary References
