# Supplementary material for: The positive piezoconductive effect in graphene
Source: Nat Commun. 2015 Sep 11;6:8119. doi: 10.1038/ncomms9119 (PMC4579395; doi:10.1038/ncomms9119)
Supplement: Supplementary Information — Supplementary Figures 1-11, Supplementary Table 1, Supplementary Notes 1-5 and Supplementary References [file ncomms9119-s1.pdf]

Supplementary Figures

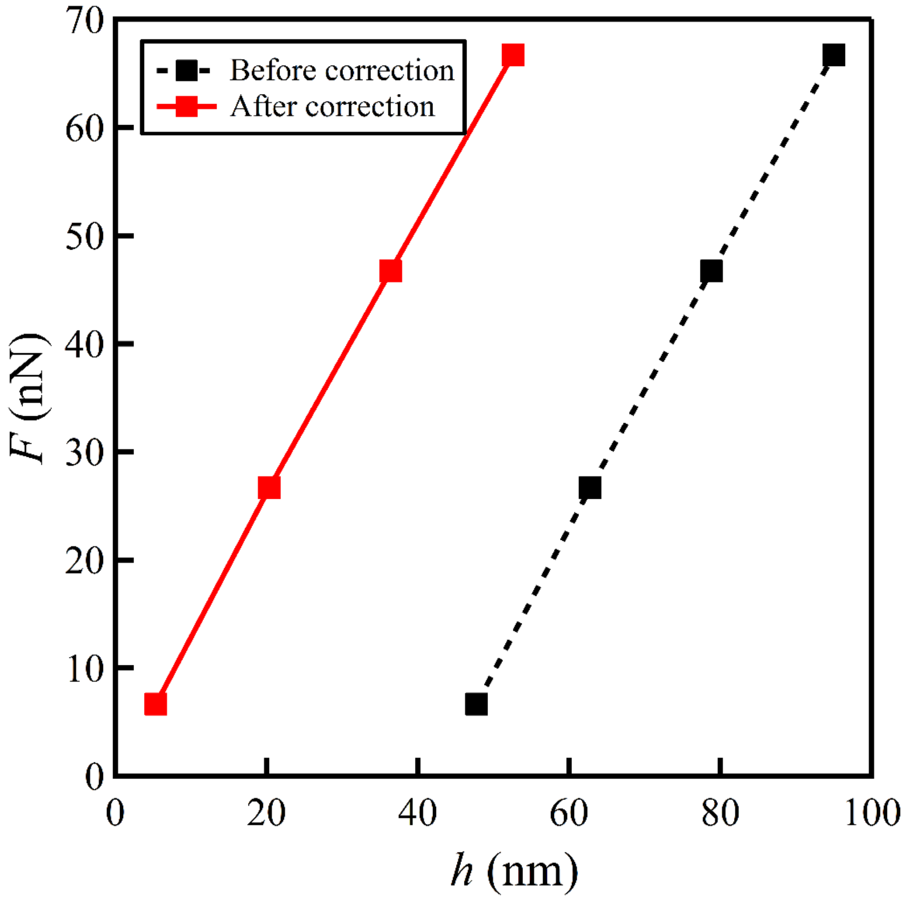

Supplementary Figure 1: Load versus elastic deformation of a typical device. The black squares represent the original data points, and the red squares represent the data points after subtracting the intercept.

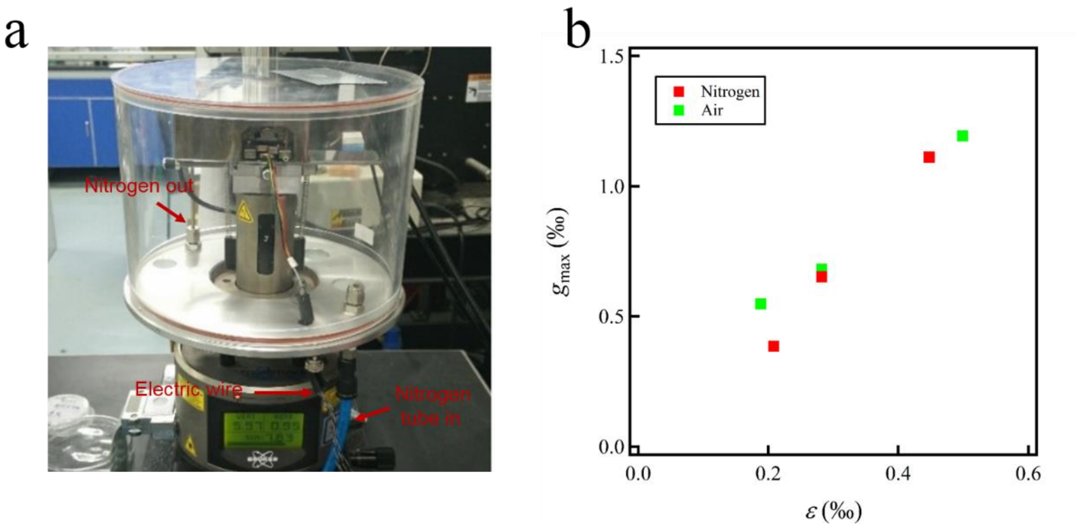

Supplementary Figure 2: Piezoconductive effect experimented in nitrogen and air. The maximum relative conductance change g_{max} as a function of strain ε for trilayer graphene. The experimental data acquired in nitrogen and air were shown by red and green squares respectively.

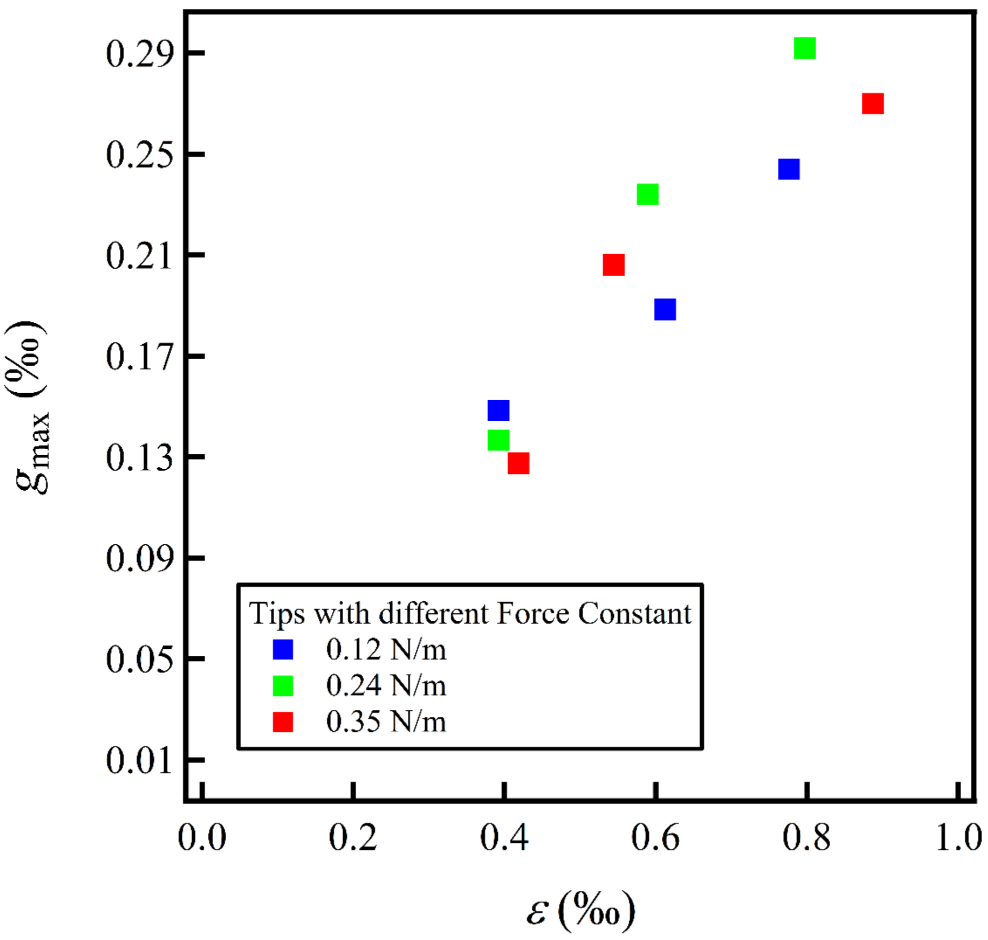

Supplementary Figure 3: Positive piezoconductive effect for three different kinds of AFM tips. The maximum relative conductance change g_{max} as a function of strain ε for a bilayer graphene. The experimental data for three different kinds of tips were shown by different color markers.

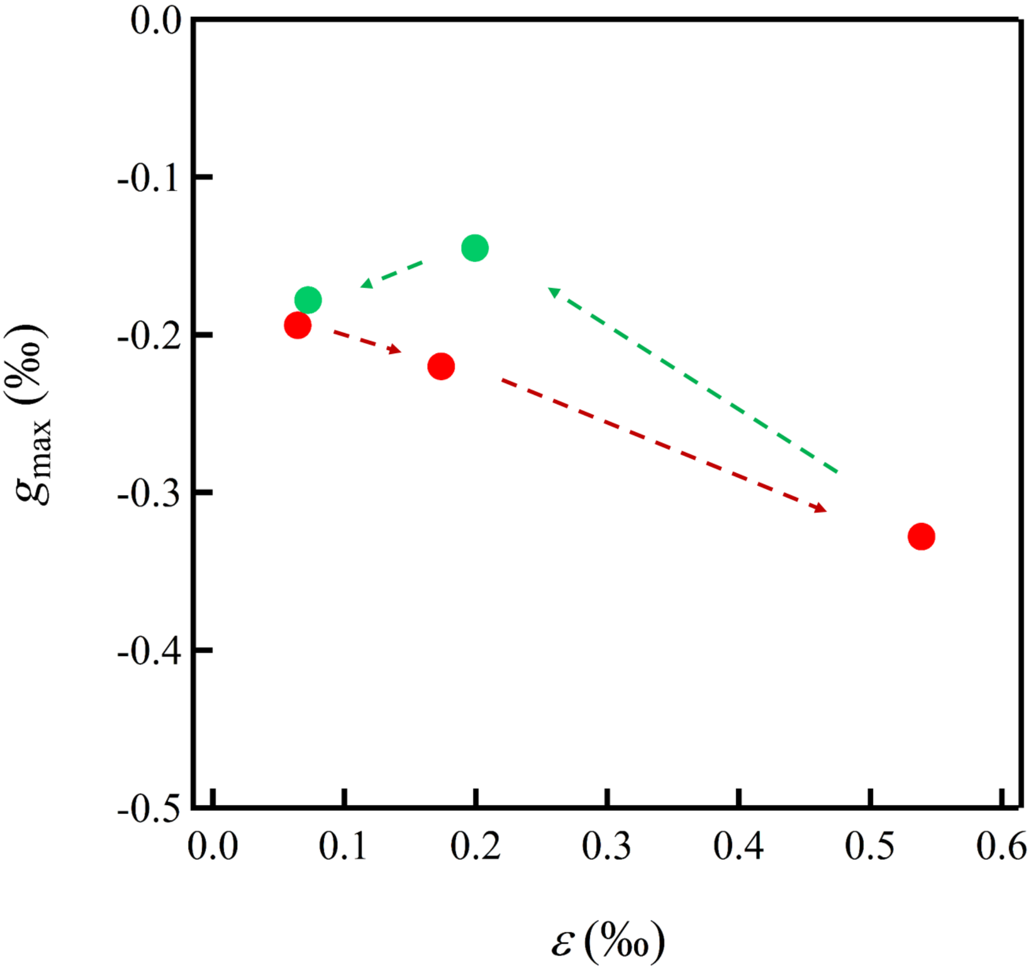

Supplementary Figure 4: Replot of g_{max} as a function of strain ε for trilayer graphene data in Figure 2e displayed in the main text. The measurement order is labelled near the data points and green dots represent the data from the reverse measurement.

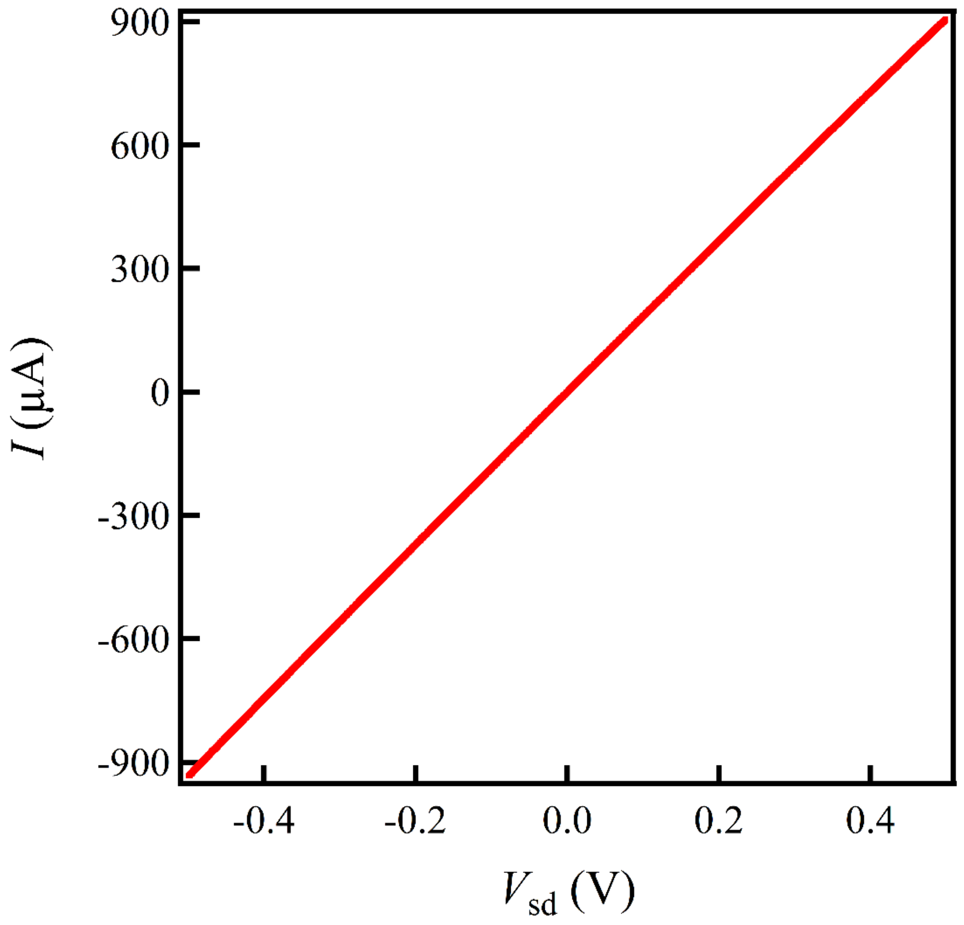

Supplementary Figure 5: *I-V* curve of the trilayer graphene device. V_{sd} is varied from -0.5 V to 0.5V.

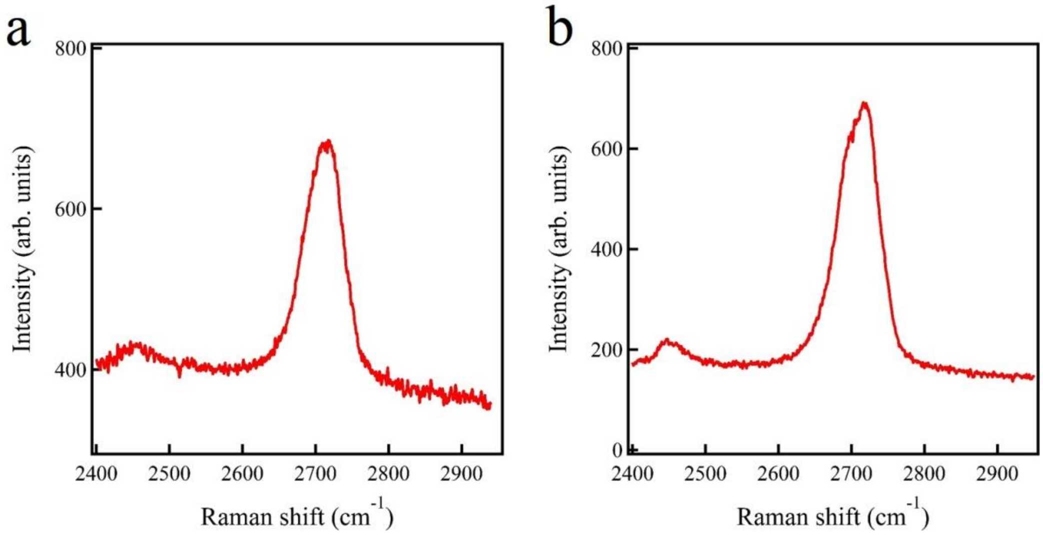

Supplementary Figure 6: Raman spectra of the tetralayer (a) and hexalayer graphene (b).

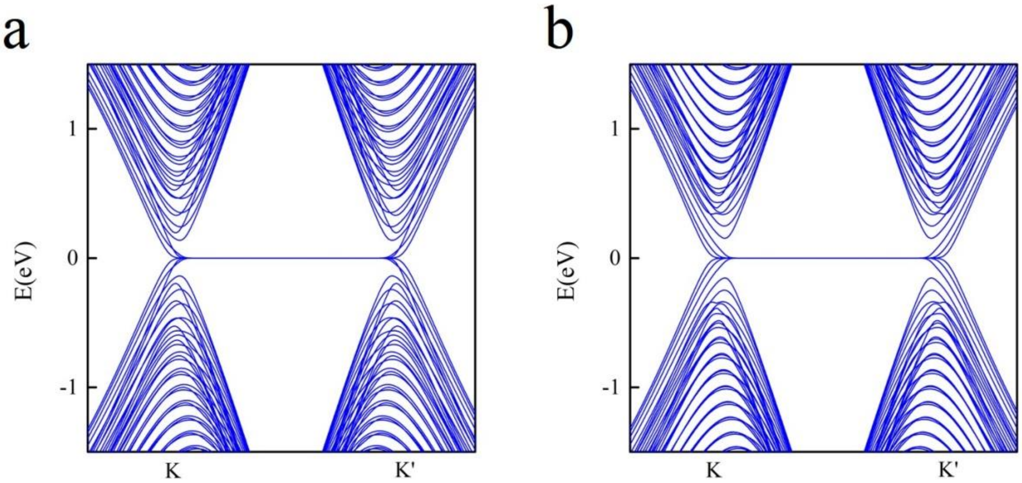

Supplementary Figure 7: Band structure of trilayer graphene nanoribbons with different stacking order. (a) ABA stacked trilayer graphene nanoribbons; (b) ABC stacked trilayer graphene nanoribbons.

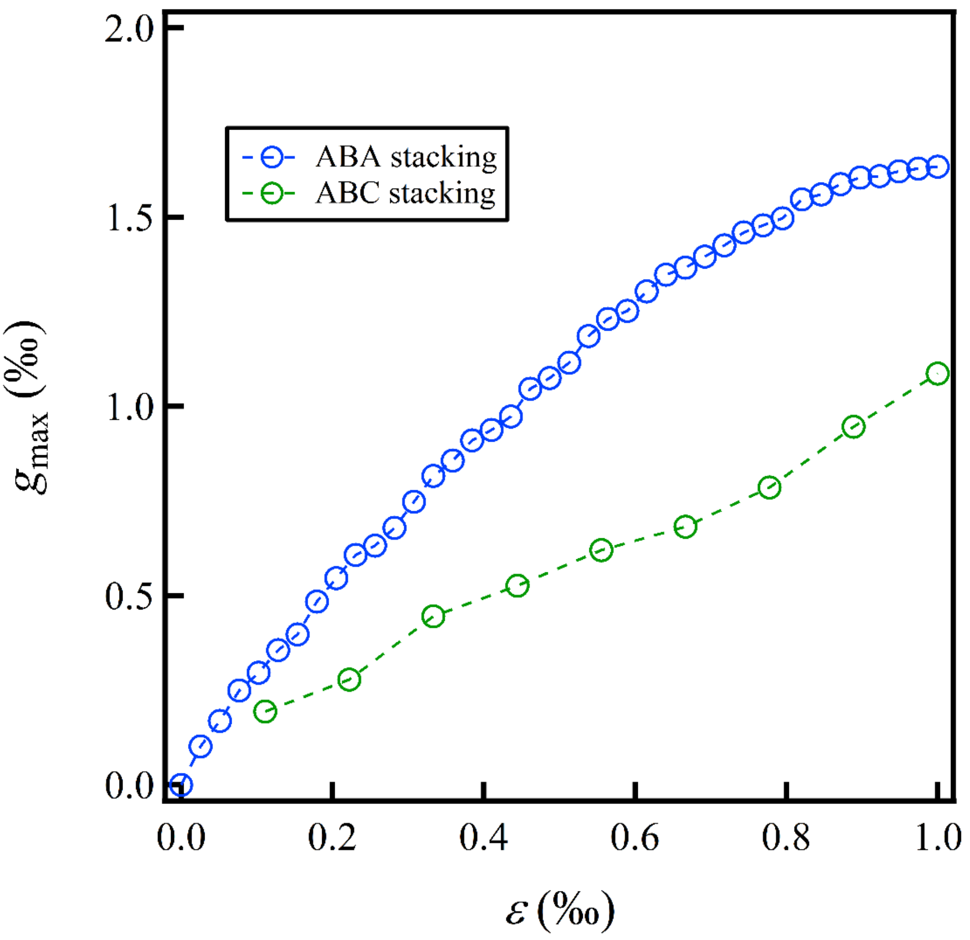

Supplementary Figure 8: The maximum relative conductance change g_{max} as a function of strain ε for ABA and ABC stacked trilayer graphene.

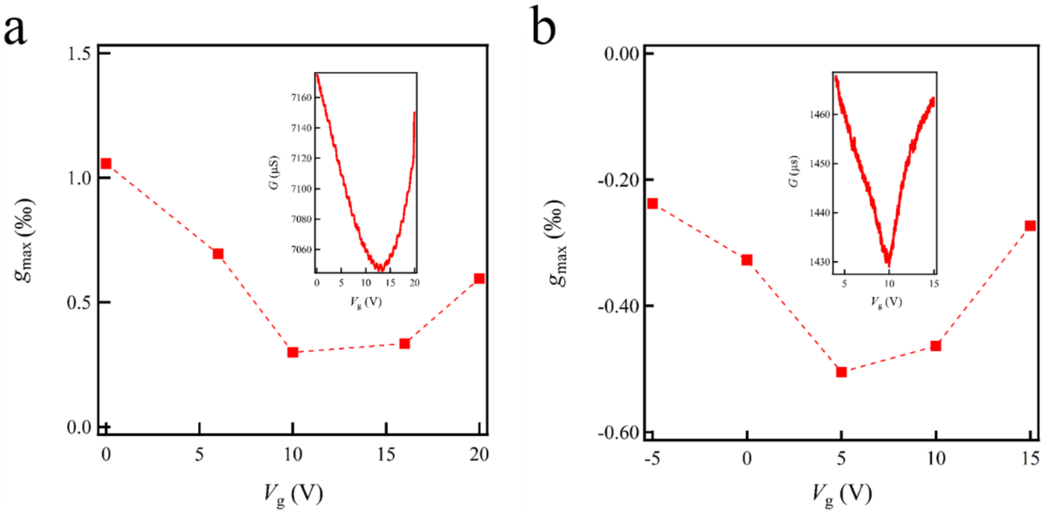

Supplementary Figure 9: Dependence of positive piezoconductive effect on back gate voltage of trilayer (a) and monolayer (b) graphene device. G versus V_g data are inserted.

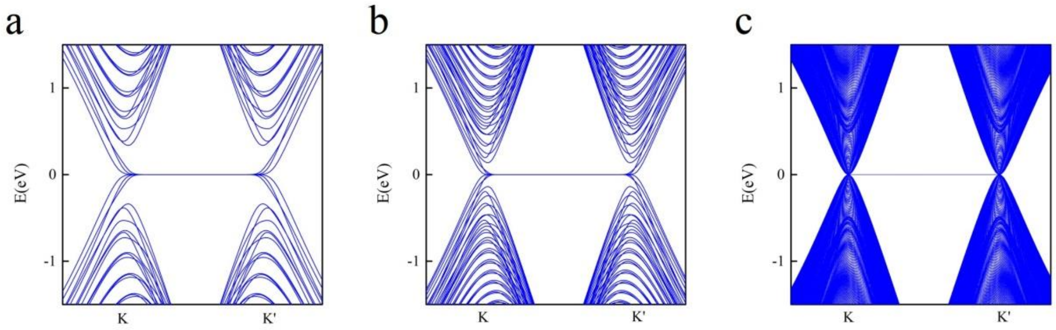

Supplementary Figure 10: Comparisons of the band structures of the zigzag trilayer (ABA stacking) graphene nanoribbons with different widths, i.e., 6 nm (a), 13 nm (b), and 100 nm (c).

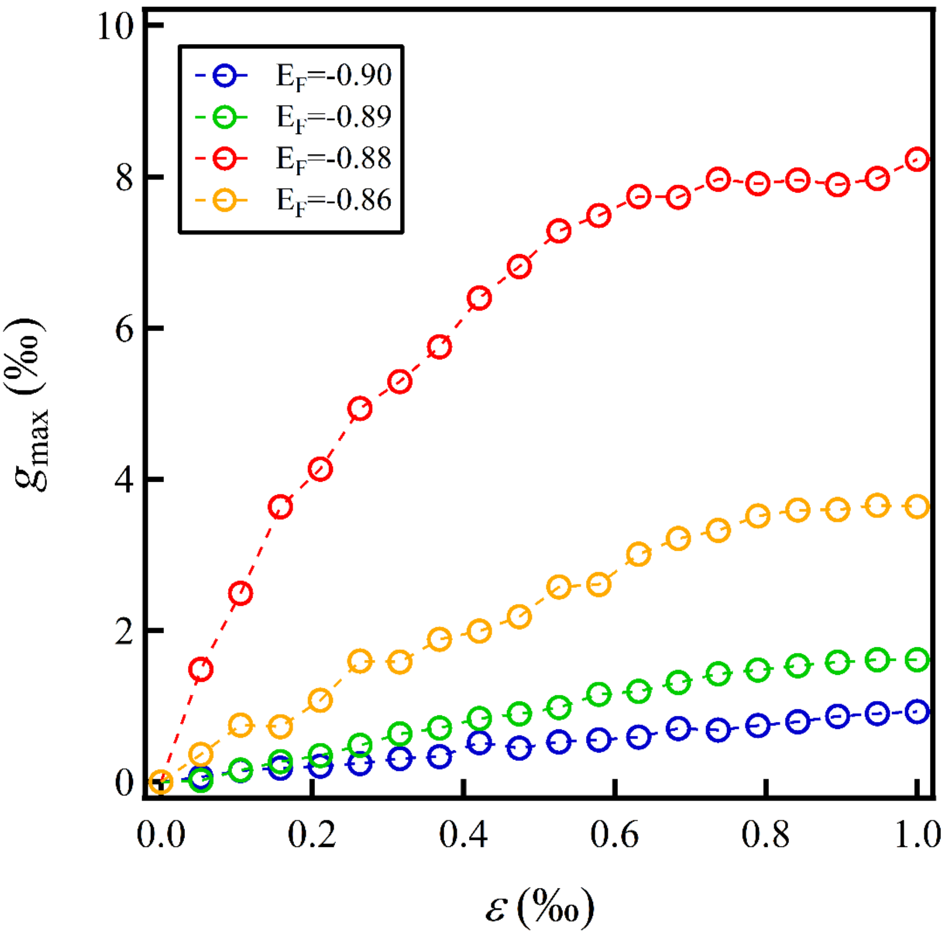

Supplementary Figure 11: Piezoconductive effects *vs* different Fermi levels for trilayer graphene. Scattering area is 13 *nm* × 100 *nm*.

Supplementary Tables

Supplementary Table 1: Fitting parameters for different layer number. $U = \eta\varepsilon$ and $\delta t = \chi\varepsilon$ in units of eV, with the fitting parameters η and χ for the bi-layer, tri-layer and the top three layers when the layer number $n > 3$ (both terms of other layers are assumed to be zero for simplicity).

n	2	3	4	5	6
η	20	15	15	15	15
χ	200	50	50	50	50

Supplementary Notes

Supplementary Note 1: The suspended bridge model in small strain regime

As described in the suspended bridge model [1], the dependence of the load *F* on the maximum strain-induced deflection *h* can be expressed by:

$$F = \frac{8wE^{2D}}{l^3}h^3 + \frac{8w\sigma^{2D}}{l}h \quad (1)$$

where w is the width, E^{2D} is the two-dimensional Young's modulus, σ^{2D} is the residual tension, *h* is the maximum strain-induced deflection, and *l* is the length of the suspended graphene. In the small strain regime, the load *F* is dominated by the second term. Experimentally, *F* can be calculated based on the deflection voltage value set in the contact mode of AFM. A typical measured *F*-*h* curve is shown in Supplementary Figure 1 (black dots). The clear linear dependence indicates the validation of the suspended bridge model in our experimental setup. The slope is proportional to the residual tension σ^{2D}, which is found to be quite small in our devices, with typical values ranging between 0.02N/m and 0.14N/m.

The intercept at the *x*-axis equals to the height of the tip at which the force begins to rise. For the devices studied, the intercept varies between 6 nm and 42 nm, which

may be attributed to the randomness of the mechanical exfoliation process. To get the precise strength of the strain, the actual maximum strain-induced deflection *h* needs to be corrected by subtracting this height. As shown in Supplementary Figure 1, the intercept is determined to be about 42nm, and the corrected *F*-*h* curve is denoted by the red dots.

Supplementary Note 2: Role of external effects

Considering our experimental setup, it is important to explore the role of several external effects that could affect the experimental observations. The details are discussed in the following:

Influence of ambient air

Our measurements were performed in ambient air, which may induce device stability issue in certain circumstances. We have investigated the influence of ambient air on the measurements very carefully. We first added an atmospheric hood (an accessory of the Bruker Multimode 8 AFM we used) to the setup of the pressure-modulated conductance microscopy (PCM), with a photograph shown in Supplementary Figure 2a. The piezoconductive measurements were then performed on a same tri-layer device in both the ambient air and pure nitrogen environment. The positive piezoconductive effect was observed in both cases, with the obtained data shown in Supplementary Figure 2b. It clearly shows that the data acquired from the pure nitrogen environment (red square symbols) are nearly collapsed with those acquired in the ambient air (green square symbols). This strongly indicates that the random doping from the external environment has negligible influence on our results.

AFM tip-induced parasitic capacitor effect

In our consideration, the graphene membranes are in contact with a non-conductive AFM tip which could induce parasitic capacitor effect. If the tip is randomly charged (*i.e.*, full of random locally charged sites), it would form a

capacitor with the graphene sheets, which would affect the carrier density and result in a graphene resistivity change. In order to explore the influence of such effect, we have repeated the piezoconductive measurements on a same bi-layer device, but using three different types of AFM tips. These tips have various force constants (0.12N/m, 0.24N/m, and 0.35N/m respectively), distinct tip geometries and therefore random charge distributions. The experimental results are displayed in Supplementary Figure 3. The observed positive piezoconductive effect follows a same curve, indicating that the tip-induced parasitic capacitor effect is also negligible in our experiments.

Local tearing

If there is a local tearing, the resistance of the suspended graphene would increase, which could affect the piezoconductive measurements (especially in the case of single layer graphene). To exclude that local tearing plays a role in our measurements, the data of the monolayer graphene in Figure 2e of the main text were taken in a measurement of hysteresis. In Supplementary Figure 4, we have clearly marked the order of the data points taken in the measurement, where two data points (green symbols) were obtained during the reverse measurement. Since the resistance change is reversible, it is reasonable to conclude that the local tearing does not occur during our measurement. Furthermore, during our measurements, for each line scan, we also monitored the conductance chance of the device in real time before and after applying the stress. If there is a local tearing due to the deformation from the AFM tip, the resulting conductance should not be able to return to its original value (due to a resistance increase) when the AFM tip re-approaches the electrodes. As shown in Figure 2a of the main text, one can find that the conductance line trace of the monolayer graphene is rather symmetric, which indicates that there is no local tearing during our measurements.

Self-heating effect

The self-heating effect may also influent the conductivity of graphene. In our experiment, we have applied a rather small source-drain bias (<0.1V) on the graphene devices by using a Lock-In Amplifier. The self-heating effects should be negligible in our measurement. To rule out this effect, we have tuned the source-drain voltage

difference from -0.5V to 0.5V to measure the current of a typical tri-layer graphene device. As displayed in Supplementary Figure 5, the I-V curve shows a linear characteristic, providing a strong evidence to exclude the self-heating effect.

Supplementary Note 3: Role of the stacking order

The Raman spectra of the four- and six-layer graphene devices are displayed in Supplementary Figure 6a and 6b respectively. After comparing with the literatures [2], we can conclude that the four-layer graphene is stacked in an ABAB order. However, it is unclear of the stacking order of the six-layer graphene device based on the obtained Raman spectra, due to a lack of related studies in literatures.

In order to explore whether the stacking order plays a crucial role in the piezoconductive effect of the multi-layer graphene, we plot the band structures of both the ABA- (a) and ABC-stacked (b) tri-layer graphene nanoribbons in Supplementary Figure 7, which are totally different [3]. Nevertheless, based on the analysis of the physical origin of the positive piezoconductive effect, we believe that the ABC-stacked tri-layer graphene should have the similar positive piezoconductive effect because of the strain-induced alterable interlayer interaction. In Supplementary Figure 8, we have theoretically verified that the ABC-stacked tri-layer graphene can also exhibit a similar positive piezoconductive effect.

Supplementary Note 4: Back-gated suspended graphene

Back-gated suspended graphene device is a rather complicated system when experiencing the combined influences from the back gate, the strain and the geometric deflection. These parameters influent the conductivity of the graphene flake jointly since they are correlated with each other. In an ideal case, tuning the back gate only

shifts the Fermi level of the whole system. However, in the real case, applying a back gate voltage on a suspended graphene device induces inhomogeneous carrier distribution and many parasitic effects due to the non-negligible geometric change. One example is the parasitic capacitive gating effect, i.e. the carrier density in the graphene increases as the graphene membranes are pushed closer to the back gate oxide during the experiments. Another example is the attractive electrostatic force-induced deflection effect [4] which alters the strain, the geometric deflection, and thus the parasitic capacitive gating effect as well. Thus, in such a gated suspended graphene system, it is highly challenging to distinguish the role of the varying Fermi level from that of these parasitic effects.

Experimentally, we have repeated the PCM experiments by applying different back gate voltages on both trilayer (see Supplementary Figure 9a) and monolayer graphene devices (see Supplementary Figure 9b). As displayed in the inset of Supplementary Figure 9a, the charge neutrality point of the tri-layer graphene is about 13V. We then measured the piezoconductive effect of this device under different back gate voltages (*i.e.*, Vg =0, 6, 10, 16, and 20V), and obtain the corresponding g_{max}. One can observe that the positive piezoconductive effect becomes more pronounced when Vg is away from the charge neutrality point, and the relation of g_{max}-V_g follows a parabolic character. For the monolayer graphene device, the experimental results show a similar parabola-shaped dependence as displayed in Supplementary Figure 9b. Such results suggest that for the higher gate voltages (negative and positive), the parasitic capacitive gating effect enhanced the conductivity, resulting in more pronounced positive piezoconductive effect in tri-layer graphene, or weakened negative piezoconductive effect in monolayer graphene.

We have also theoretically investigated the piezoconductive effect as a function of the Fermi level, and found that the finite-size effect plays a significant role in determining the relationship between the piezoconductive effect and the Fermi level. There is a detailed discussion in the following note "**Supplementary Note 5: Finite size effect in the theoretical calculations**".

Supplementary Note 5: Finite size effect in the theoretical calculations

In our numerical calculations, we have considered a system size of 13 *nm* × 100 *nm* (about 3×10^5 atoms for trilayer graphene), and our numerical results are able to qualitatively explain the experimental findings. However, due to the limitation of the computational capacity, it is impossible to model an extremely large system that is comparable to the practical devices. Therefore, it is unavoidable to introduce the finite size effects in the numerical calculations. In Supplementary Figure 10, we plot the band structures of the zigzag trilayer graphene nanoribbons with different ribbon widths, *i.e.*, 6 *nm* (a), 13 *nm* (b) and 100 *nm* (c). One can see that there is a completely distinct energy spectra around the charge neutrality point (*i.e.*, $E_F=0$ in our calculation). The weakness of the finite size effect can be solved by setting the Fermi level to be slightly away from the charge neutrality point E_F=0 in our numerical calculations, and the difference of the energy spectra near the charge neutrality point does not qualitatively affect our results. It is noteworthy that all the samples in our experiments are p-doped, leading to the fact that the Fermi level is naturally away from the charge neutrality point.

In addition, the number of transverse modes for a given Fermi level of the small systems (*e.g.* 6 *nm* and 13 *nm*) is significantly different from the large systems (*e.g.* 100 *nm*). As displayed in Supplementary Figure 11, the maximum relative conductance change g_{max} is highly dependent on the Fermi level due to the density of transverse modes is closely rely on the Fermi level in the numerical calculations. However, one can clearly observe that the piezoconductive effects for different Fermi levels are all positive in the tri-layer graphene. This shows that our numerical calculation can be able to qualitatively capture the physical origin of the positive piezoconductive effect. In our numerical calculations, we have better explained the experimental findings by choosing suitable parameters (*e.g.* the Fermi level). However, we want to stress that our theory just aims to qualitatively reveal the physical origin of the piezoconductive effect, but not to exactly fit the experimental data.

Supplementary References

1. Herbert, E.G., Oliver, W.C., De Boer, M.P. & Pharr, G.M. Measuring the elastic modulus and residual stress of freestanding thin films using nanoindentation techniques. *J. Mater. Res.* **24**, 2974-2985 (2009).
2. Nguyen, T.A., Lee, J., Yoon, D. & Cheong, H. Excitation Energy Dependent Raman Signatures of ABA-and ABC-stacked Few-layer Graphene. *Sci. Rep.* **4**, 4630 (2014).
3. Sanvito S., Lambert C. J., Jefferson J. H. & Bratkovsky A. M. General Green's-function formalism for transport calculations with *spd* Hamiltonians and giant magnetoresistance in Co-and Ni-based magnetic multilayers. *Phys. Rev. B* **59**, 11936 (1999).
4. Bao W., Myhro K., Zhao Z., Chen Z., Jang W., Jing L., Miao F., Zhang H., Dames C. & Lau C. N., In Situ observation of electrostatic and thermal manipulation of suspended graphene membrane. *Nano Lett.* **12**, 5470-5474 (2012).
